# STOP using just GO: a multi-ontology hypothesis generation tool for high throughput experimentation

**DOI:** 10.1186/1471-2105-14-53

**Published:** 2013-02-14

**Authors:** Tobias Wittkop, Emily TerAvest, Uday S Evani, K Mathew Fleisch, Ari E Berman, Corey Powell, Nigam H Shah, Sean D Mooney

**Affiliations:** 1 Buck Institute for Research on Aging, Novato, CA, USA; 2 University of Michigan Medical School, Ann Arbor, MI, USA; 3 National Center for Biomedical Ontology, Biomedical Informatics, Stanford University, Stanford, CA, USA; 4 Department of Medical and Molecular Genetics, Indiana University School of Medicine, Indianapolis, IN, USA

## Abstract

**Background:**

Gene Ontology (GO) enrichment analysis remains one of the most common methods for hypothesis generation from high throughput datasets. However, we believe that researchers strive to test other hypotheses that fall outside of GO. Here, we developed and evaluated a tool for hypothesis generation from gene or protein lists using ontological concepts present in manually curated text that describes those genes and proteins.

**Results:**

As a consequence we have developed the method Statistical Tracking of Ontological Phrases (STOP) that expands the realm of testable hypotheses in gene set enrichment analyses by integrating automated annotations of genes to terms from over 200 biomedical ontologies. While not as precise as manually curated terms, we find that the additional enriched concepts have value when coupled with traditional enrichment analyses using curated terms.

**Conclusion:**

Multiple ontologies have been developed for gene and protein annotation, by using a dataset of both manually curated GO terms and automatically recognized concepts from curated text we can expand the realm of hypotheses that can be discovered. The web application STOP is available at http://mooneygroup.org/stop/.

## Background

High throughput experimentation such as gene expression microarrays, next generation sequencing or proteomics enables the interrogation of many thousands, or even millions, of data points simultaneously. Comparison between these experiments (such as a phenotype and control) enables identification of gene or protein sets of interest in a hypothesis free manner. To stimulate generation of testable, explanatory hypotheses for experimental validation from these sets of genes, researchers will often apply Gene Set Enrichment Analysis (GSEA) [1] or concept enrichment analysis using controlled vocabulary terms. Term enrichment analysis, which refers to the search for ontology terms that occur more in a given gene list when compared with a background gene set, can be used to generate new scientific hypotheses. Gene Ontology (GO) [2,3], arguably the most commonly used ontology in basic research, consists of a collection of three non-overlapping controlled vocabularies that describe molecular functions, biological processes and cellular components. There are now more than 50 GO-based enrichment analysis tools available. Examples of such functional analysis tools are BiNGO [4] or GOEAST [5] , which solely utilize gene ontology (GO) for their analyses. Other approaches, such as ClueGO [6], DAVID [7] and GeneWeaver [8], incorporate larger range of sources, such as disease ontologies, phenotype ontologies or common pathways. However, all of them rely on predefined gene annotations and thus are limited to biomedical domains that have curated annotations. Baumgartner, *et al.*[9] presented an analysis that demonstrated how manually curated annotations can never keep pace with novel scientific discoveries, and argued that text-mining based methods need to be adopted to keep pace with the rising volume of literature. For example, an incredible amount of established knowledge about genomes and proteomes is available through NCBI Entrez Gene [10] and UniProt [11], but the concepts mentioned in the textual descriptions of genes and proteins in these resources are not part of any statistical enrichment analysis. We believe in a hybrid approach of testing manually curated terms along with automatically recognized concepts from curated text will result in more hypotheses and therefore be more useful to the researcher.

Large-scale annotation of all the known genes and expressed proteins in an organism’s genome is a complex and arduous task. To this end, biology and medicine have created and manage discipline-specific structured ontologies that are suitable for gene or protein annotation. Although these ontologies are publicly available, for instance via the National Center for Biomedical Ontology [12,13] or the EBI Ontology Lookup Service [14,15] and provide valuable information about connections between different biological concepts, only a small fraction of these ontologies are used for gene and protein annotation and therefore a relatively small amount of annotations are actually available for use in enrichment analysis methods.

The quality of results from term enrichment analysis is naturally dependent on the quality of the annotations underlying the analysis. Therefore term enrichment analysis should only use high quality annotations, such as the human-curated annotations from Ingenuity Pathway Analysis (IPA) (http://www.ingenuity.com/) or from a highly restricted subset of GO of experimentally validated and published annotations. However, many genes do not have annotations of this quality, and therefore the results of enrichment analysis can be highly incomplete. On the other end of the spectrum, including automated annotations based on criteria such as computational prediction using sequence similarity would result in a richer but less accurate set of annotations and hence less reliable results from term enrichment analysis. In this paper, we propose a middle ground that combines high quality human-curated gene descriptions with automated assignment of annotation terms based on those descriptions. We use the Stanford National Center for Biomedical Ontology (NCBO) Annotator [16], which provides annotations with terms from over 200 publicly available biomedical ontologies, to automatically annotate a gene or protein based on the corresponding Entrez Gene or UniProt textual description. The text description is used as the basis on which the NCBO Annotator provides ontological terms that could annotate the gene or protein

We find that automated annotations generated in this manner reliably recover the known annotations already present in the text record (such as GO terms or OMIM [17] terms), and we find that we are able to annotate with a wide spectrum of concepts not available in any currently used ontology enrichment tools. Additionally, we are able to identify GO terms that are present in curated text that are not currently formerly annotated to these genes or proteins, and many of these examples are bona fide annotations. Overall, our approach is able to annotate proteins with 524,304 terms from across 291 ontologies; and a vast majority of these terms are not part of the GO.

In the following, we will demonstrate the advantages of using automatic annotations that are based on manually curated textual descriptions, by extending our previous RANSUM approach [18] to enable analysis of genes and protein concepts. We will first describe the STOP workflow, which allows a researcher fast and easy statistical analyses of gene sets using up-to-date information of genes and proteins from the most widely used model organisms and human. We will further demonstrate how automatically derived annotations contain valuable information that is not currently present in the GO, without diminishing the value of manually curated GO enrichment analysis. Therefore, we compare our annotations against GO and highlight examples of gene-to-term annotations that are likely to be correct but not present in official GO annotations. Finally we describe two use-cases: (1) proteins that are direct protein interaction partners of the huntingtin protein and (2) known Parkinson’s disease genes. We use these sets of proteins to demonstrate how STOP can reveal interesting enriched concepts that improve the understanding of functional traits implied by gene sets.

## Results

### Term enrichment with automatically derived annotations

Here, we present STOP (Statistical Tracking of Ontological Phrases), a web resource that utilizes automatic annotation and term enrichment analyses to generate novel insights into common traits of sets of genes. In contrast to commonly used tools for the task of gene set enrichment analysis, STOP does not limit itself to predefined annotations and a few controlled vocabularies, but uses up-to-date information (curated text) about genes to map them to terms from all 291 ontologies provided by the National Center for Biomedical Ontology (NCBO). Results from an analysis with STOP annotations are presented in a web interface that allows easy navigation and identification of concepts that summarize the input set of genes; thus helping researchers interpret and understand experimental results to create novel hypotheses.

**Figure 1:**
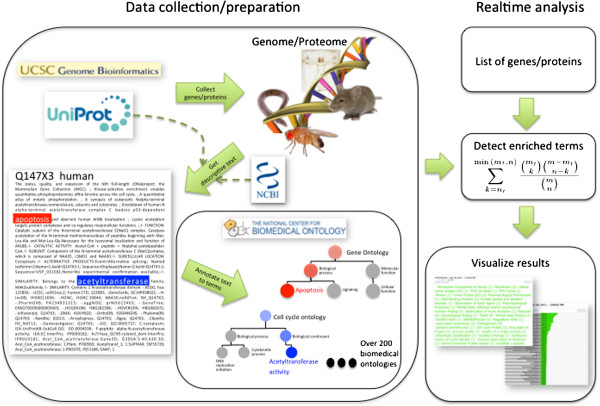
**Overview of the computational workflow of STOP. **The left side illustrates the backend of the STOP software, i.e. the automatic annotation pipelin: (1) The genome and proteome of all included species is retrieved from UniProt and Entrez Gene respectively and subsequently (2) descriptions for all genes/proteins are collected from UniProt and Entrez Gene and finally (3) submitted to the NCBO annotator web service. The information is stored in a MySQL database and can be accessed by the frontend, which is displayed here on the right side. The real-time analysis pipeline requires a list of genes as input and calculates for each term of the 200+ ontologies whether it is enriched in the given gene list. The results are subsequently presented as a tag cloud or in list form

The computational pipeline underlying the STOP backend as well as the real time enrichment analysis provided by the STOP frontend via a web interface are explained graphically in Figure 1.

We implemented a fully automatic import process that can be executed at very frequent intervals (currently once a month) to always provide the latest, state-of–the-art information about genes. This process of populating our local database includes:

1. Collecting all genes and proteins in the genome/proteome of the 6 widely used model organisms *Mus musculus*, *Rattus* norvegicus, *Drosophila melanogaster, Caenorhabditis elegans*, *Saccharomyces cerevisiae* and *Escherichia coli* and *Homo sapiens* via UniProt and NCBI Entrez Gene .

2. Search, download and filtering of descriptive text for each gene/protein using NCBI Entrez Gene and UniProt.

3. Annotation of the genes/proteins to terms from all ontologies currently present in the NCBO Bioportal database, using the Annotator Web service.

STOP currently uses over 667,258,930 annotations of 226,298 genes and 200,047 proteins from 7 organism (*H. sapiens, M. musculus, R. norvegicus, D. melanogaster, C. elegans, S. cerevisiae,* and *E. coli*) that come from 524,304 terms across 291 ontologies. The included ontologies can provide general information as in GO and NCI Thesaurus [19], or can be more specific as, for instance, in the Disease Ontology [20], Pathway Ontology [21], or Human Phenotype Ontology [22].

STOP has been optimized for fast processing to compute and display enriched terms from 291 ontologies in a matter of seconds. The resulting term list can still be overwhelming. Therefore, we: (i) Remove redundant information by combining terms with the same name; (ii) Implement filtering methods that can display results from selected ontologies, ontology categories, or terms that match user specific key words; (iii) Identify a list of 41 highly informative ontologies that can be accessed as “Preferred” using the ontology category filter; (iv) Visualize the enriched terms as a sorted table or term cloud. We further ease usage of our web interface by utilizing all gene identifier mappings for genes and proteins as available from NCBI Entrez Gene and UniProt respectively. To use STOP a researcher simply has to: (1) copy + paste their list of genes or protein (delimited via whitespace, comma, semicolon, tab or newline), (2) select the species, the background set of genes and the multiple-hypothesis correction method from drop-down menus, and (3) press the submit button. The STOP web interface is available at http://mooneygroup.org/stop.

### Comparing gene ontology with automatic annotations

As GO is still the standard ontology when analyzing gene sets for their functions, we compared the official GO annotations to those we automatically derive within STOP for this ontology. We obtain the gold standard GO annotations for proteins from UniProt and for genes from Entrez gene (gene2go file). We further compared gene-based annotations with annotations from the respective species-specific databases: GOA [23] (human), FlyBase [24] (fly), WormBase [25] (worm), RGD [26] (rat), SGD [27] (yeast), EcoCyc [28] and MGI [29] (mouse) obtained via http://www.geneontology.org. The results are slightly lower in Recall and Precision, probably due to database differences between Entrez Gene and the species-specific sites. In this analysis we consider each gene in our background that is also found in GO, *i.e.* genes that have at least one GO term annotation. For each such gene we calculate precision and recall and determine the average of these values to evaluate the overall equality between these two approaches. The precision for one specific gene is the ratio of GO terms that are annotated in both the gold standard and our annotation to that gene (true positives) divided by all annotations that we predict for this gene (true positives + false positives). The average precision for an organism is the average of all precision for all genes in that organism. As expected we achieve high recall values ranging from 0.96 to 1 for the different species except E.coli. When comparing our annotations with E.coli we see big differences, which seem to be rooted in the limited GO annotations that are present in Entrez Gene (our annotation source) for E. coli. The generally high recall values are due to the fact that GO terms are part of the text that comprises our input for the annotation process. Missing terms are easily explained by different versions and changes in GO. The lower precision shows that STOP finds several novel annotations that are currently not in GO and thus are counted as false positives in this evaluation. We find however, that along actual false positives many of these new annotations make sense and probably should be included in GO, see below for some examples. For the annotation process we integrated GO annotations from UniProt and Entrez Gene while the comparison has been performed on the most recent version obtained from UniProt GOA, Entrez Gene and http://www.geneontology.org/.

The results (presented in Table 1) show that for most genes we identify already known GO terms and add several annotations that are not present in the manual annotations. We found several examples where genes or proteins have functions associated with them that are only described in UniProt/Entrez Gene but are not yet associated with relevant GO terms. One example is the human protein liver carboxylesterase 1 (P23141). STOP associates this protein with the GO term ‘cocaine metabolic process’ (GO:0050783). This association is not listed on the GO annotations website. This association was identified from a title for a reference paper for the protein, “Structural basis of heroin and cocaine metabolism by a promiscuous human drug-processing enzyme” [30]. Another example can be found in the *C. elegans* protein (Q27539) ATP-dependent Clp protease proteolytic subunit 1, mitochondrial. STOP annotated this protein with the GO concept mitochondrial unfolded protein response’ (GO:0034514), however this concept was not in the GO annotations. The concept was identified from one of the references associated with this protein, “ClpP mediates activation of a mitochondrial unfolded protein response in *C. elegans*” [31].

**Table 1:** Summary of comparison between STOP and GO annotations

**Species**	**Annotation source/gold standard**	**Recall**	**Precision**	**F-measure**
**human**	Entrez Gene/Entrez Gene	0.993	0.678	0.806
	Entrez Gene/GOA	0.979	0.674	0.798
	UniProt/GOA	0.998	0.608	0.756
**mouse**	Entrez Gene/Entrez Gene	0.990	0.791	0.879
	Entrez Gene/MGI	0.990	0.791	0.879
	UniProt/GOA	0.999	0.746	0.854
**rat**	Entrez Gene/Entrez Gene	0.987	0.724	0.835
	Entrez Gene/RGD	0.959	0.713	0.818
	UniProt/GOA	0.999	0.736	0.847
**fly**	Entrez Gene/Entrez Gene	0.987	0.767	0.863
	Entrez Gene/FlyBase	0.978	0.762	0.857
	UniProt/GOA	0.992	0.751	0.855
**worm**	Entrez Gene/Entrez Gene	0.998	0.783	0.878
	Entrez Gene/WormBase	0.998	0.783	0.878
	UniProt/GOA	0.999	0.788	0.881
**yeast**	Entrez Gene/Entrez Gene	0.994	0.798	0.885
	Entrez Gene/SGD	0.994	0.798	0.885
	UniProt/GOA	0.998	0.630	0.773
**E. coli**	Entrez Gene/Entrez Gene	1.000	0.611	0.758
	Entrez Gene/EcoCyc	0.340	0.354	0.347
	UniProt/GOA	0.964	0.826	0.890

Annotations based on Entrez Gene descriptions are compared against the gene2go annotations from Entrez Gene and species-specific databases where the annotations have been downloaded from http://www.geneontology.org, and STOP annotations based on UniProt descriptions are compared against GOA annotations. Recall and Precision are calculated for each gene and subsequently averaged. The F-measure is the harmonic mean of these average Recall and Precision values.

### Using STOP to improve understanding of Huntington’s disease

**Figure 2:**
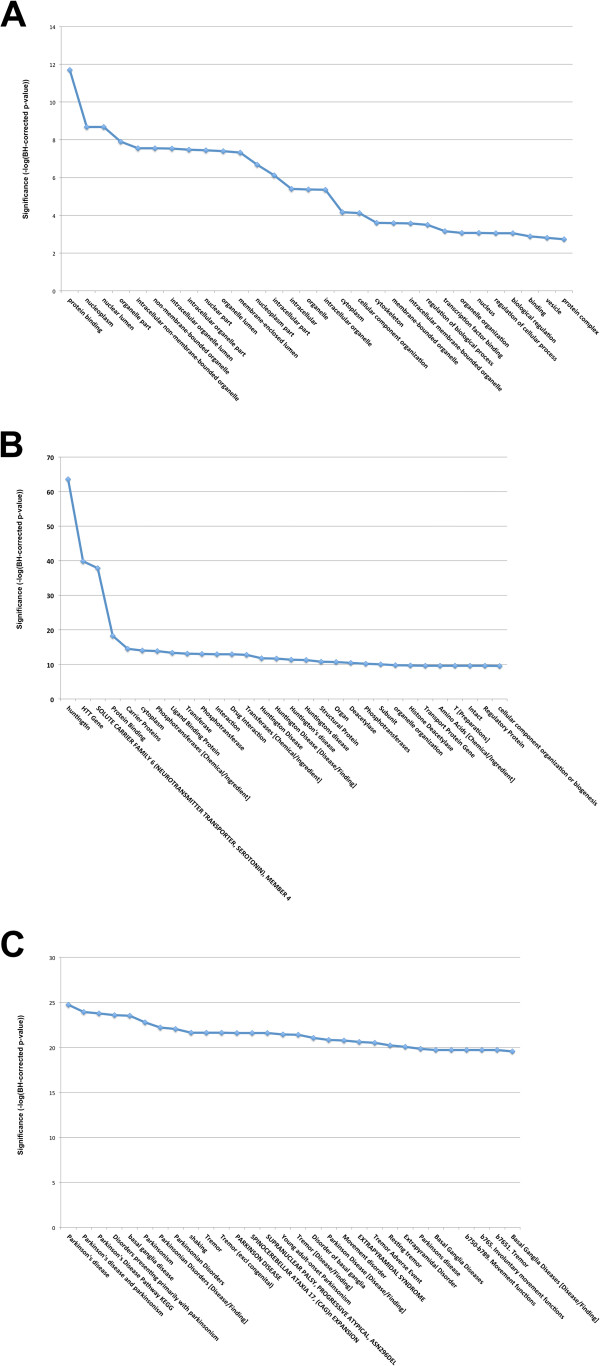
**Top 30 enriched terms for DAVID and STOP analysis of Htt interacting proteins and STOP analysis of Parkinson’s genes. **Fifty-nine genes from the HPRD database known to interact with the Human Huntingin (HTT) gene were analyzed using STOP and DAVID (GO). 14 proteins known to be involved in Parkinson’s disease were analyzed with STOP. (**A**) The list of HTT interacting proteins was submitted to DAVID, and enrichment analysis carried out with GO_all using SwissProt Human as the background. The top 30 annotations are shown. (**B**) The same proteins were also submitted to STOP with the same background, and the results were limited to annotations from the preferred ontologies. (**C**) The Parkinson’s related proteins were similarly analyzed with STOP; again limited to annotations from the preferred ontologies. The top 30 categories are shown along with their significance. Significance is defined as the –log(Benjamini-Hochberg corrected p-values). For reference, p = 0.01 is equivalent to 2

In order to assess the functional utility of STOP, we selected a set of proteins from the Human Protein Reference Database (HPRD) that are known to directly interact with the human Huntingtin gene (HTT) [32]. HTT is of particular interest in neurodegeneration because it is prone to polyglutamine expansion, the degree of which correlates to the severity of the development of Huntington’s disease, a devastating neurodegenerative disease. The list of interacting proteins, which is stored on the gene level in HPRD, consists of 59 genes (excluding HTT) serves as a test case for STOP here (see Additional file 1). Since the interactions are on the protein level UniProt/SwissProt IDs were used in the analysis, and the SwissProt Human database was used as the background for the enrichment analyses. As an additional point of comparison, the same list of proteins was submitted to DAVID and all enriched gene ontology (GO) annotations were retrieved using the “GO_all” database. The analysis using DAVID returned a typical list of enriched GO categories (Figure 2A). Among the terms that can be associated directly with what is known about Huntington’s disease were for example protein complex assembly, induction of apoptosis and terms associated with cell death. Biologically, each of these terms describes at least some part of what little is known about the function of HTT. However, these (and the other terms) don’t give much information about the gene set as a whole. If, for instance, one were to submit this gene list not knowing how or if the genes had any shared biological relevance, the results of the GO enrichment analysis would be difficult to interpret and would likely not contribute to the understanding of the dataset.

When the HTT PPI gene list was analyzed using STOP, the results were more diverse. For the purposes of this test case, the results of the STOP enrichment analysis were filtered using only terms from the “Preferred” ontologies (see Additional file 2), which helps to refine the output to more biologically useful annotations (Figure 2B). In this case, the top 30 enriched annotations include terms similar to those in the GO analysis such as protein binding or cellular component organization. However, the results also include more descriptive terms such as Huntington’s Disease, huntingtin, Transferases, drug interaction, and solute carrier family 6 (neurotransmitter transporter, serotonin). Thus, the STOP analysis correctly identified this gene list as being associated with Huntington’s disease, neuron-related processes, and specific disease pathways (histone deaceltylases). It is important to note that the HTT gene was not a part of the submitted gene list in either analysis.

### Application of STOP to Parkinson’s disease

To further validate the utility of STOP, we applied it to genes and proteins associated with Parkinson’s Disease (PD). All proteins in UniProt that are associated with PD were identified in the PhenoPred resource [33], resulting in 14 human proteins (see Additional file 3). STOP was applied on this list resulting in many enriched terms using Benjamini-Hochberg for correction of multiple hypotheses and UniProt/SwissProt as background (Results for the top 30 enriched categories in the preferred set of ontologies can be seen in Figure 2C). Not surprisingly the top term was Parkinson’s Disease (p < 1.87 × 10^-25^), which was found in 20 ontologies (6 preferred ontologies). Other terms included Basal Ganglia Diseases (9.86 × 10^-21^), Tremor (2.39 × 10^-22^), Movement Disorders (1.50 × 10^-17^), Substantia Nigra (1.24 × 10^-16^), Brain Diseases (3.02 × 10^-13^), Age (7.79 × 10^-14^), Dopamine (5.74 × 10^-12^), Neuron (4.80 × 10^-11^), and many others. All terms appear to be relevant, with possible false positives been related largely to other neurodegenerative diseases such as Alzheimer Disease (1.20 × 10^-19^) or Spinocerebellar Ataxia 17, (CAG)n Expansion (2.60 × 10^-22^). Less significant are terms associated with pathology but are too general to be useful such as Lab (8.59 × 10^-9^) or Patients (9.88 × 10^-7^).

## Discussion

Some ontological annotations don’t make scientific sense, for example, human genes and proteins that are automatically annotated with terms from the *C. elegans* Phenotype Ontology. Given that, the philosophy of our approach was to capture the widest possible number of term annotations, in a hypothesis-free manner, regardless of the source. Since all examination of these annotations is through statistical enrichment, we believe that falsely discovered annotations will not be statistically enriched after multi hypothesis correction. Users, of course, are allowed to select out any term sets in real time on the website, preventing the presence of spurious terms.

Using automated annotations derived from text can, however, lead also to false positive annotations. An example we observed, was that results for protein sets that were obtained from interaction data often had the terms, “mint”, “menthol”, and “vascular plant” enriched. We could trace this to the interaction database MINT [34] which was part of the descriptive text of all proteins, that had an interaction stored in that database. As a consequence we exclude common database names from the gene descriptions. On the other hand, unrelated terms can have a true meaning that can only be detected with automated text-based methods. In an analysis of a set of genes that are involved in Parkinson’s disease, we observed the term “Australia” as enriched (using Entrez Gene, p < 3.03 × 10^-8^). Originally assuming this to be a false positive we identified the source as a research group in Australia that is leading in Parkinson’s research due to a highly cited manuscript with Australia mentioned in the title. Although not biologically relevant, this example shows that automated annotations are capable of detecting relations that would otherwise remain undetected.

Due to the import process, the STOP application completely depends on the ontologies that are made available through and the annotator web service that is provided by NCBO. However, the NCBO constantly expands their database of available ontologies and adds mapping information between terms of different ontologies. STOP takes advantage of this growing resource by regularly re-annotating (about once a month) the list of genes with up-to-date gene descriptions.

Interestingly, we found that in many cases proteins would be annotated with GO terms that were not found in the gold standard GO annotation database from the GO consortium website. We pursued this and found that these annotations were often correct. While this is out of the purview of the STOP method, it suggests that curators would do well to identify term text and fold them into their own annotations.

## Conclusion

We have constructed a tool that substantially broadens the hypotheses that can be generated with enrichment analysis using automatically created annotations. We find that these annotations are able to identify existing known concepts in the text. Users can download our species specific annotation datasets and perform enrichment analysis on our website with a list of gene and protein IDs. Enriched and depleted terms can be filtered by ontology or ontology type. Furthermore, annotations can be downloaded into a spreadsheet for later use. In the end, STOP enables experimental research projects to identify hypotheses for gene and protein sets using a concept space that is far larger than GO or OMIM, thereby improving their ability move high throughput experimentation to validation.

## Methods

### Automated annotation pipeline

In order to build the necessary components to perform enrichment analysis, the following was performed. First, a list of the genes and proteins for each genome and proteome was compiled using Entrez Gene and UniProt. Using a web service, the text descriptions for each gene or protein were collected. This text was then used as input into the NCBO Automated Annotator, where ontological concepts were annotated upon that text. This is then repeated for each list of genes and proteins for each species, including human, mouse, fly, nematode worm, rat, yeast and E. coli. Detailed description of these steps are below.

1. **Collect whole genome/proteome gene/protein lists**

The first step is to identify all genes/proteins in the genome/proteome of all species. The genes in a genome are determined using the Entrez Gene database and the proteome is similarly determined by UniProt using all proteins that contain the “whole proteome” keyword. We provide subsets of the genomes/proteomes as predefined background for the statistical analysis: (1) all Entrez Gene genes, (2) only RefSeq reviewed/validated genes, and (3) only protein coding genes as gene backgrounds and (1) UniProt/Swissprot and (2) UniProt/Tremble as protein backgrounds. However, we annotate all genes/proteins in the genomes/proteomes as described in the subsequent steps.

2. **Collect descriptive text for each gene/protein**

There are several publicly available databases that provide information about genes and proteins. The text descriptions for genes are downloaded from the FTP site of the NCBI Entrez Gene database and the text descriptions for proteins are downloaded from the UniProt database. The Entrez Gene text is downloaded as binary file and converted into XML format The descriptive text for proteins is obtained in TXT format from UniProt. We extract valuable information from both resources while removing unnecessary informations such as author names that could lead to false positive annotations. For Entrez Gene the descriptive text includes a gene summary, short descriptive texts from GeneRIF and known annotations and interactions. For UniProt the text for each protein has a summary that describes the proteins’ function, a list of publication titles that are associated with a protein and already known annotations and keywords. We store the type of text (e.g. gene summary, GeneRIF, or publication title) and the text itself to being able to add evidence to obtained annotations in future releases of STOP.

3. **Annotate concepts upon text**

There are several publicly available databases that provide information about genes and proteins. The text descriptions for genes are downloaded from the FTP site of the NCBI Entrez Gene database and the text descriptions for proteins are downloaded from the UniProt database. The Entrez Gene text is downloaded as binary file and converted into XML format The descriptive text for proteins is obtained in TXT format from UniProt. We extract valuable information from both resources while removing unnecessary informations such as author names that could lead to false positive annotations. For Entrez Gene the descriptive text includes a gene summary, short descriptive texts from GeneRIF and known annotations and interactions. For UniProt the text for each protein has a summary that describes the proteins’ function, a list of publication titles that are associated with a protein and already known annotations and keywords. We store the type of text (e.g. gene summary, GeneRIF, or publication title) and the text itself to being able to add evidence to obtained annotations in future releases of STOP.

3. **Annotate concepts upon text**

All text describing genes and proteins is read by the NCBO Automated annotator. The NCBO annotator uses a library of terms and their synonyms from over 200 biomedical ontologies. It applies the string matching algorithm MGrep on our input text and finds all exact matches of available term names or known synonyms thereof in the submitted text. It filters known stopwords such as “the”, “and”, “is” etc. and each annotation is propagated to the root, i.e. if a text is annotated to a term it is automatically annotated to all its parents following the “is_a” relationship in the respective ontology. The available parameters of the NCBO Annotator are specified in Additional file 4 list of available ontologies which we can annotate to, are listed in Additional files 2. Note, that although the NCBO provides mapping between terms across ontologies, we decided to leave out this option to allow for a more independent annotation of each ontology.Since each input text is associated with a gene/protein we obtain annotations for genes/proteins from the annotator. Subsequently, we simply remove redundant annotations and store each annotation in a local database which then can be accessed by our web frontend and analysis backend. An example of this workflow is shown in Figure 1.

### Computing enrichment analysis

We apply the most widely used hypergeometric test to identify concepts that are overrepresented with respect to a background set of genes. A user may choose to provide such a set, or use one of our predefined background sets for genes ((1) Entrez Gene, (2) RefSeq reviewed/validated, of (3) protein-coding) or proteins (UniProt/Swissprot or UniProt/Tremble). All analyses are done separately for each ontology, i.e. multiple hypothesis correction is done on an individual ontology basis and only those terms with at least one input gene annotated to it are analyzed and contribute to the multiple hypothesis corrections. Let in the following n denote the number of genes in our study and m the number of genes in the background with at least one annotation in the respective ontology, i.e. genes that have no annotation in that ontology are ignored. Further let n_t_ and m_t_ be the number of genes annotated to a term in the study set and the background respectively. The p-value *p(t)* representing the likelihood that a term *t* has annotations to at least as many genes as we observe in our list of genes is calculated using the one-tailed version of Fisher’s exact test [35], also known as hypergeometric test:

$$p\left(t\right)=\sum_{k=n}^{\min\left(m_{t},n\right)}\frac{\left(\begin{array}{l} m_{t}\\ k \end{array}\right)\left(\begin{array}{l} m-m_{t}\\ n-k \end{array}\right)}{\left(\begin{array}{l} m\\ n \end{array}\right)}$$

In order to correct for multiple hypotheses, a user may choose between the three most commonly used methods Bonferroni [36], Bonferroni-Holm [37], and Benjamini-Hochberg [38]. STOP applies this correction per individual ontology to guarantee consistent results that are independent of the user’s choice of ontologies. A term is reported as enriched if the adjusted p-value is below a significance threshold of 0.05. STOP reports only terms that have a significant corrected p-value and at least 3 genes annotated to it.

### Implementation

**Figure 3:**
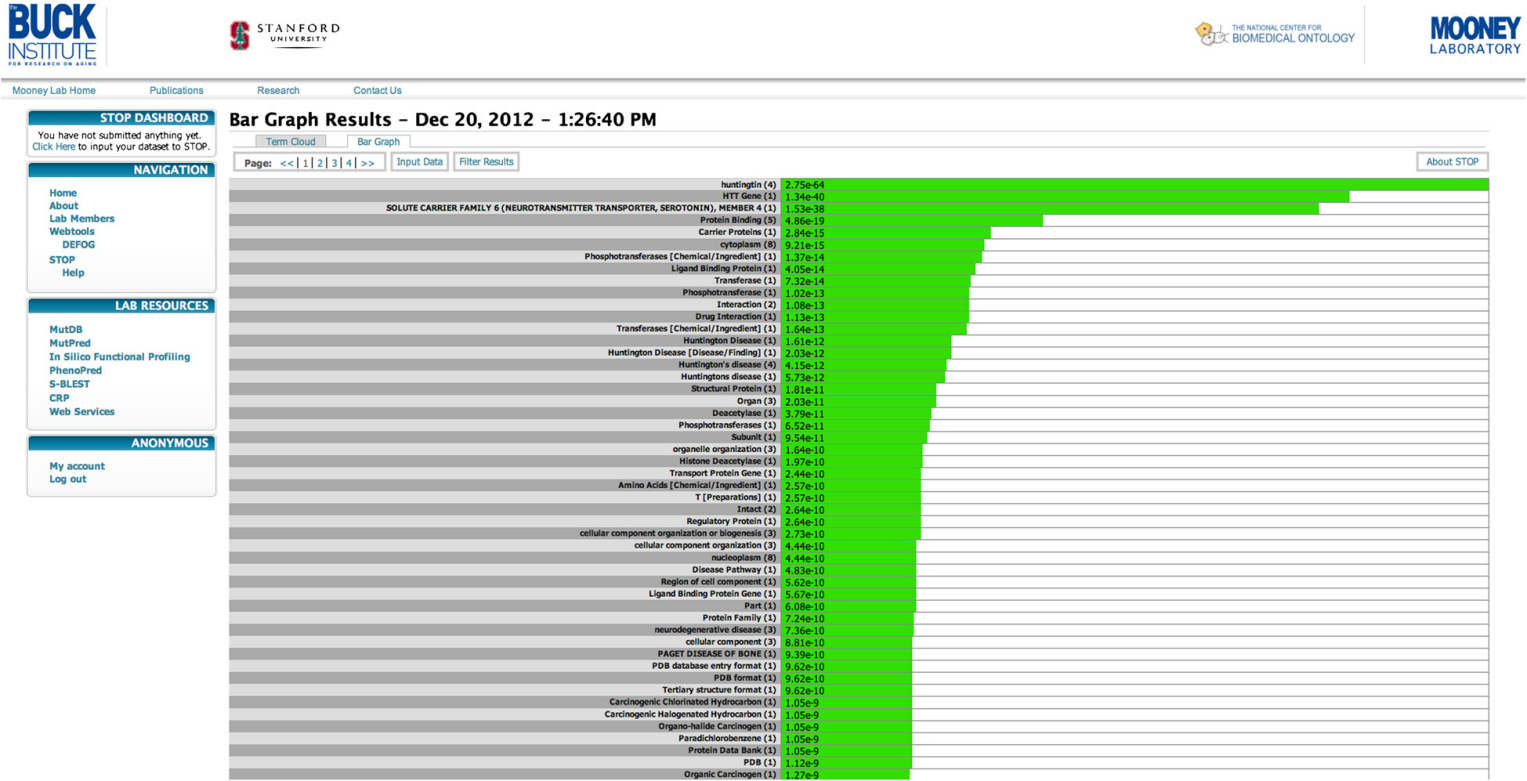
**The STOP website showing results in a bar graph. **On the left the navigation interface with previously performed jobs is shown and on the right the enriched categories for the Huntingtin primary interactors that are present in our list of preferred networks are displayed

The STOP website was constructed using DRUPAL and requires a user to submit an email address or create an account. All annotations are stored locally in a MySQL database and the enrichment analysis back-end as well as the import process have been implemented in JAVA. A job usually finishes in under a minute (Figure 3).

## Competing interests

The authors declare that they have no competing interests.

## Authors’ contribution

TW, ET, UE, and KMF implemented the software. CP assisted with the statistical questions. AEB, ET and TW evaluated the method and created use-cases. NS added support from NCBO, a crucial part within the STOP method. SDM supervised the whole project and initiated the idea. All authors contributed to writing the manuscript. All authors read and approved the final manuscript.

## Supplementary Material

Additional file 1 **List of proteins from the Human Protein Reference Database **(HPRD) that are known to directly interact with the human Huntingtin gene (HTT). Click here for file

Additional file 2 **List of all ontologies that are available for the NCBO annotator web service. **The table lists all ontologies that are available for the NCBO annotator. Ontologies of our “preferred” category are highlighted in red. Click here for file

Additional file 3 **List of proteins associated with Parkinson’s Disease (PD). **All proteins in UniProt that are associated with PD were identified in the PhenoPred resource. Click here for file

Additional file 4 List of all parameters for the NCBO annotator and the values that were used in STOP. Click here for file
